# Novel *GLDC* Compound Heterozygous Variant Leading to Nonketotic Hyperglycinemia: Case Report and Literature Review

**DOI:** 10.3389/fped.2021.725930

**Published:** 2021-08-27

**Authors:** Yanyan Cao, Lingzhi Meng, Yudong Zhang, Jiancheng Jiao, Weicong Pu, Li Ma

**Affiliations:** ^1^Institute of Pediatric Research, Children's Hospital of Hebei Province, Shijiazhuang, China; ^2^Department of Neonatology, Children's Hospital of Hebei Province, Shijiazhuang, China

**Keywords:** nonketotic hyperglycinemia, *GLDC* variation, compound heterozygous variant, glycine cleavage enzyme system, inherited metabolic disease

## Abstract

Nonketotic hyperglycinemia (NKH) is a lethal autosomal recessive disease resulting from alterations in glycine metabolism, commonly caused by mutations in glycine decarboxylase (*GLDC*). The symptoms of NKH usually manifest in the neonatal period, and can be categorized into severe NKH and attenuated NKH based on the clinical outcome. To date, only a few NKH cases have been reported in China. We here report a case of a neonate with severe NKH carrying a novel compound heterozygous variant in *GLDC*. The patient was a 68-h-old girl who had progressive lethargy, no crying, and poor sucking ability from birth, and was therefore transferred to our department. On admission, the patient was supported by intubation and ventilation and presented with profound coma. Metabolic investigation indicated a markedly increased glycine concentration both in the plasma and cerebrospinal fluid (CSF). Symptomatic treatments were administered, but the patient's condition did not improve substantially. Whole-exome sequencing identified compound heterozygous mutations (c.1261G>C, p.G421R and c.450 C>G, p.N150K) in *GLDC*, which were inherited from the mother and the father, respectively. The patient was hospitalized for 8 days in our department and died 2 days after discharge. We further summarize the clinical features, genetic characteristics, administered treatment, and prognosis of previously reported Chinese NKH patients for context. Our results highlight that due to the non-specific clinical phenotypes of NKH and difficulty in obtaining CSF samples, genetic testing is a crucial tool, not only for a diagnosis but also for predicting the clinical outcome and can potentially help to determine the optimal therapeutic strategy.

## Introduction

Nonketotic hyperglycinemia (NKH) is an autosomal recessive inherited metabolic disease characterized by deficient activity of the glycine cleavage enzyme system (GCS), leading to the accumulation of glycine in almost all body tissues. The GCS consists of four components: glycine decarboxylase (also known as P protein), aminomethyl transferase (T protein), hydrogen carrier protein (H protein), and dihydrolipoamide dehydrogenase (L protein), encoded by *GLDC, AMT, GCSH*, and *DLD*, respectively. *GLDC* mutations account for ~80% of NKH cases, whereas *AMT* variants account for ~20% of cases (1). In the majority of NKH cases, symptoms first appear in the neonatal period or during early infancy. According to the clinical outcomes, NKH is classified into severe NKH and attenuated NKH. Approximately 85% of cases with neonate onset are classified as severe NKH (2), mainly presenting with progressive lethargy and marked hypotonia, as well as severe apnea that requires ventilation.

We here report the case of a neonate with NKH carrying a novel compound heterozygous variant in *GLDC*, and summarize the clinical and genetic features of children with NKH reported in China to date.

## Case Description

A 68-h-old girl presenting with lethargy and a poor nutritional state among other symptoms was transferred from a local hospital to our department. She was born at 37 + 3 weeks of gestation by cesarean section due to cephalopelvic disproportion from a gravida 1, parity 1 (G1P1) mother. She had a birth weight of 2,600 g, an Apgar score of 10, and did not present asphyxia. Her parents were healthy and non-consanguine. After birth, the child manifested with progressive lethargy, no crying, and poor sucking ability. She was admitted to a neonatal department in the local hospital at 40 h of life, where she showed low spirit, decreased spontaneous breathing, poor terminal circulation, and blood pressure of 39/22 mmHg. She was intubated and ventilated, and administered 0.9% sodium chloride and dopamine (12 μg·kg^−1^·min^−1^) intravenously, as well as cefoperazone. However, the patient's condition did not substantially improve, and she was therefore transferred to our department.

On admission, physical examination showed profound coma with intubation and ventilation, yellowish skin, no autonomic activity, no autonomic breathing, no response to orbital pressure stimulation, slow pupil response to light, soft limbs, and poor peripheral blood circulation. Blood gas analysis showed respiratory acidosis (pH = 7.197, P_CO2_ = 65.7 mmHg, P_O2_ = 93.4 mmHg, [HCO_3_] = 24.9 mM, [Be] = 4.1 mM, and O_2_ saturation of 97.4%). Electrolytes and lactic acid levels were normal, and blood glucose was slightly high (7.8 mM).

## Diagnostic Assessment

**Figure 1:**
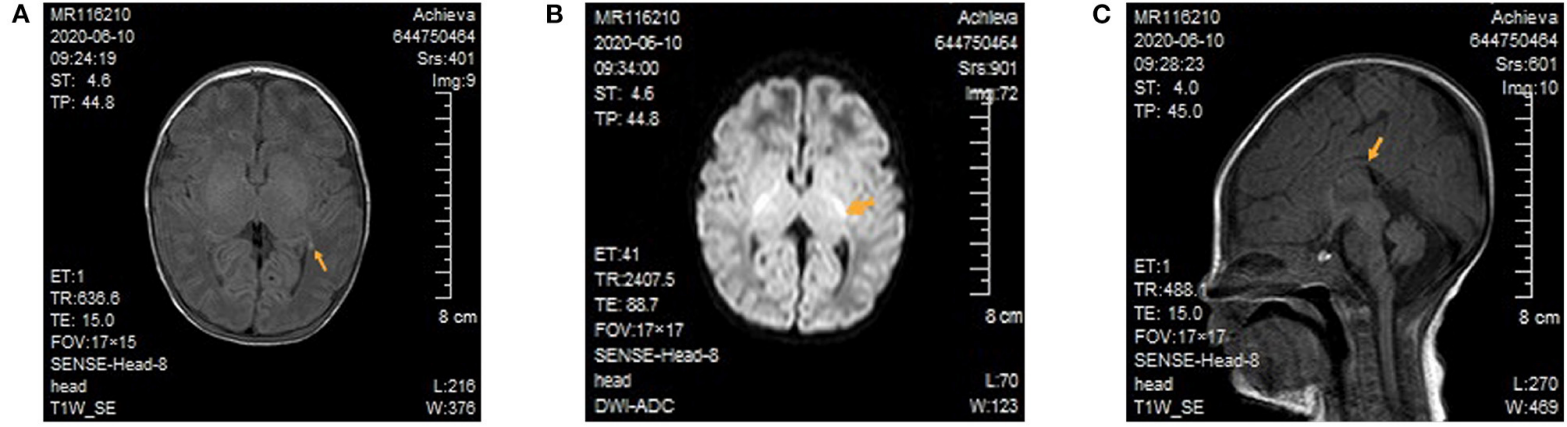
Cranial magnetic resonance imaging of the patient. **(A)** Focal cerebral hemorrhage of the left paraventricular brain. There was no high T1 signal in the posterior limb of bilateral internal capsule. **(B)** In the diffusion-weighted image, high signals can be seen for the posterior limb of the internal capsule. **(C)** The thin and short of corpus callosum.

After admission, the patient continued to be supported by intubation and ventilation, indwelling catheterization, and administration of cefoperazone and sulbactam sodium to combat any potential infection. Because a diagnosis of congenital metabolic diseases could not be excluded, a regimen including vitamin B12, vitamin B6, vitamin C, folic acid, coenzyme Q10, and levocarnitine was administered. Several subsequent blood ammonia checks were in the normal range, and there was no obvious metabolic/respiratory acidosis in the repeated blood gas analysis. Amplitude-integrated electroencephalogram both on 3 and 5 days of life all indicated a burst suppression pattern (the amplitude ranged from 1μV to nearly 100 μV), absence of sleep-wake cycle and no convulsive activity. Cranial magnetic resonance imaging (MRI) demonstrated focal cerebral hemorrhage of the right frontal lobe and left paraventricular brain (Figure 1A), focal long T2 signal of the bilateral frontal parietal lobes, and high signals of diffusion-weighted imaging of several brain areas, including the bilateral radial crown, posterior limb of the internal capsule (Figure 1B), and pontine arm, and the thin corpus callosum (Figure 1C). On day five of admission, the blood amino acid and acylcarnitine spectrum analyses to check for inherited metabolic diseases showed that the glycine concentration was increased at 942 μM (normal range 100–500 μM). The metabolic spectrum of the cerebrospinal fluid (CSF) also showed a markedly elevated glycine level, reaching a value of 3,460 times the control. The ratio of CSF to plasma glycine was 0.12 (normal <0.08). A comprehensive panel of urine organic acids was normal.

**Figure 2:**
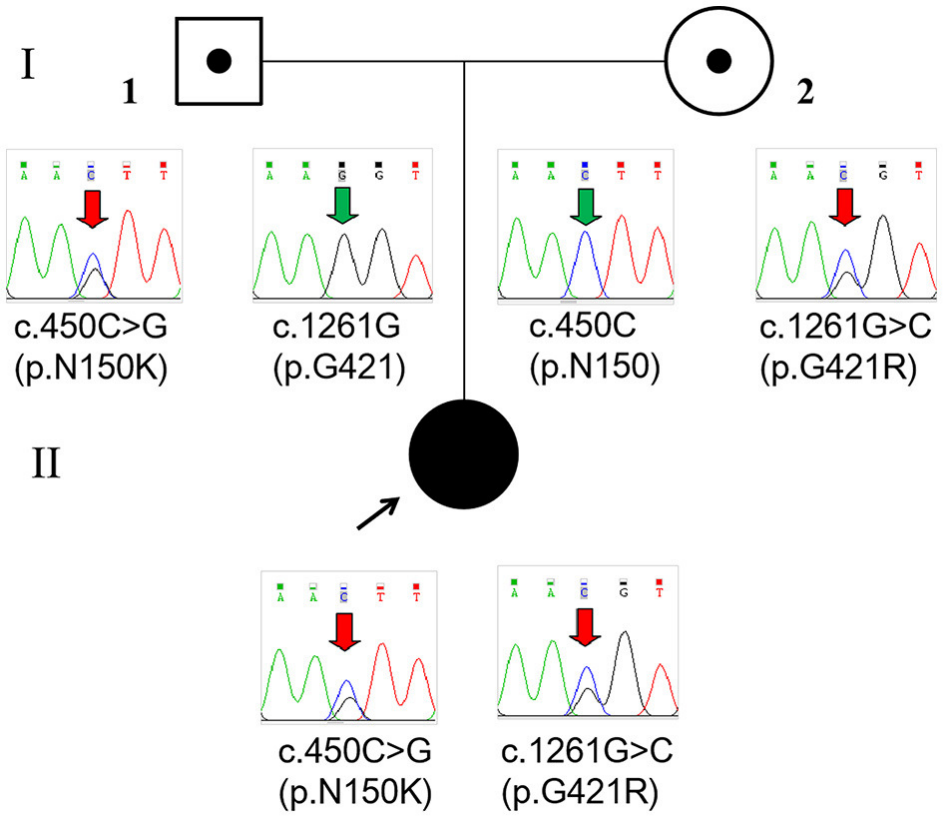
Pedigree and *GLDC* variations confirmed by Sanger sequencing of the family. The patient (II) had a compound heterozygous variation c.450C>G (p.N150K) and c.1261G>C (p.G421R), which were inherited from her father (I-1) and mother (I-2), respectively.

Whole-exome sequencing (Beijing Fulgent Technologies Inc., Beijing, China) indicated that the patient had compound heterozygous variants in *GLDC* (reference genome hg 19, NM_000170.2): c.1261G>C (p.G421R) in exon 9 and c.450 C>G (p.N150K) in exon 3, which have not been previously reported. Sanger sequencing also confirmed that the two mutations were inherited from her mother and father, respectively (Figure 2). According to American College of Medical Genetics and Genomics guidelines, these two missense mutations are classified as likely pathogenic. The two mutations were inherited from the mother and father, respectively. Mutations were absent from controls in the GnomAD, 1000 Genomes Project, Exome Aggregation Consortium, and dbSNP databases. Bioinformatic prediction analyses using Mutation_Taster, PolyPhen2, and SIFT indicated that they were deleterious. Two other two missense mutations, p.G421V and p.N150T, at the same amino acid position had been identified as pathogenic. A diagnosis of NKH was finally made based on the clinical symptoms, elevated glycine concentration in the plasma and CSF, and increased glycine CSF/plasma ratio.

After 8 days of comprehensive anti-infection treatment, mechanical ventilation, and other symptomatic care, the patient's conscious reaction, limb movement, and peripheral circulation improved, although she still had no obvious spontaneous breathing. Considering the poor prognosis, the parents ceased treatment, and the patient died 2 days after discharge.

## Discussion

Approximately 80% of the NKH-causing *GLDC* mutations are sequence variations, whereas the remainder are exonic copy number variations (CNVs) (3). Among the sequence variations, missense mutations are the most frequent, followed by nonsense mutations, splice-site mutations, and small insertions/deletions. In addition, some recurrent mutations have been identified in the United Kingdom and Finland, because of a founder effect, almost all of them unique (3, 4).

To date, 13 *GLDC* variants, including seven missense mutations (53.8%), three CNVs (23.1%), two nonsense mutations (15.4%), and one splice-site mutation (7.7%), have been identified in 10 Chinese children with NKH from 7 different families (Table 1) (5–10). Among 11 alleles identified with single-nucleotide variants, three (27.3%) were in exon 21 and two (18.2%) were in exon 9. No mutations in *AMT* have been identified in Chinese children suffering from NKH to date. In this study, we detected two heterozygous missense mutations in a neonate with severe NKH, c.1261G>C (p.G421R) and c.450 C>G (p.N150K), which were inherited from the mother and the father, respectively.

**Table 1:** Clinical characteristics and *GLDC* variants in Chinese NKH children.

**Case**	**Sex**	**Age of onset**	**Clinical presentation**	**Exons**	**Mutation**	**Inherited**	**Plasma glycine (normal range) (μmol/L)**	**CSF glycine (normal range) (μmol/L)**	**CSF/plasma ratio (normal ratio)**	**Treatment**	**Outcome**	**References**
1	Male	1 d	poor feeding and decreased activity, lethargy, hypotonia, absent deep tendon reflexes, developmental delay, myoclonic seizures	21/20	c.2516A>G (p.Y839C)/c.2457 + 2T>A	Mother/father	947.8 (115–600)	226.4 (3–20)	0.24 (<0.02)	Sodium benzoate, pyridoxine, dextromethorphan, dietary restriction of natural protein intake	Alive at 1 year 7 months: improved deep tendon reflexes and muscular hypotonia; but still poor feeding and intellectual disability	Liu S, et al. (5)
2	Male	11 h	early metabolic encephalopathy and Ohtahara syndrome	15/4–15	c.1786 C>T (p.R596X)/Exon 4–15 deletion	Mother/father	Normal	NA	NA	Adreno-cortico-tropic-hormone, topiramate and dextromethorphan	Died at 4 m	Gao Z, et al. (6)
3	Male	after birth	progressively poor reaction and weak crying	21/21	c.2198 C>T (p.A733V)	Mother/father^a^	1304.32 (130–650)	NA	NA	Antibiotics, mechanical ventilation and nutritional support	Died after 4 d	Dai H, et al. (7)
4	Male	5 d	poor reaction, feeding difficulty and limb tremor	13/9p24.3p22.3	c.1607G>A(p.R536Q)/ 9p24.3p22.3 deletion	Mother/NA^b^	1409.16 (125–750)	NA	NA	Cardiopulmonary resuscitation and trachea intubation and mechanical ventilation	Died at 6 d	Cheng L, et al. (8)
5	Male	9 m	intractable seizure	25/9	c.3006C>G (p.C1002W)/c.1256C>G (p.S419X)	Mother/father	75 (0~276)	45.3 (1.6~19.5)	0.06 ( ≤ 0.02)	NA	Alive at 6 year 8 months: intractable seizure, severe bilateral spastic paralysis and intellectual disability	Jiang T, et al. (9)
6	Female	2 year	ataxia, chorea and behavioral abnormality	25/9	c.3006C>G (p.C1002W)/c.1256C>G (p.S419X)	Mother/father	Normal	36.7 (1.6–19.5)	0.13 ( ≤ 0.02)	NA	Alive at 3 year 5 months: language retardation, ataxia, chorea and behavioral problem	
7	Male	2 d	Lethargy, hypotonia, seizures, apnea	23/3	c.2680A>G(p.T894A)/ Exon 3 deletion	Mother/father	NA	NA	NA	NA	Died at 11 d	Lin Y, et al. (10)
8	Male	2 d	Lethargy, hypotonia, seizures, apnea, hiccup	23/3	c.2680A>G(p.T894A)/ Exon 3 deletion	Mother/father	1587.87 (232-740)	260.2 (2.2-14.2)	0.164 (<0.08)	NA	Died at 13 d	
9	Female	3d	Lethargy, hypotonia, seizures, hiccup	23/3	c.2680A>G(p.T894A)/ Exon 3 deletion	Mother/father	1038.25 (232-740)	157.2 (2.2-14.2)	0.151 (<0.08)	Sodium benzoate, dextromethorphan	Alive at 7 m: severe intellectual disability, frequent seizures	
10	Female	after birth	Lethargy, no crying, poor feeding and hypotonia	9/3	c.1261 G>C (p.G421R)/c.450 C>G (p.N150K)	Mother/father	942 (100–500)	Markedly elevated	0.12 (<0.08)	Antibiotics, mechanical ventilation, vitamin B12, B6 and C, folic acid, coenzyme Q10 and levocarnitine	Died at 13 d	This study

a *The patient's sample was not obtained, and his parents carried a heterozygous mutation in GLDC*.

b *Copy number variation was not analyzed in the parents*.

*NA, Not available*.

Different types of genetic variants can lead to a complete absence or different degrees of residual GCS enzyme activity, thus leading to distinct clinical phenotypes. For example, loss-of-function mutations such as a copy number (11), frameshift, nonsense, or splice site variation (12) causing complete depletion of GCS activity can lead to a severe phenotype (3, 13–15). However, it is difficult to assess the residual GSC activity in the case of missense mutations (4, 16). In addition to the present report of a severe case of NKH, there have been seven severe cases of NKH and two cases of attenuated NKH reported in China. Among the 12 alleles associated with severe NKH (eight cases from six unique families), seven (58.3%) were missense mutations, whereas in the two alleles associated with the attenuated cases (two cases from one family), only one was a missense mutation. Although the effect of the novel mutations identified in this study, p.G421R and p.N150K, on GCS activity is not clear, this patient was diagnosed with severe NKH according to the age of onset and clinical characteristics, suggesting that both of them were null mutations. Furthermore, the same amino acid positions of *GLDC*, p.G421V and p.N150T have been previously reported in three different patients with NKH. A patient carrying the p.N150T and p.R790W mutations presented with hypotonia apnea, coma intraventricular hemorrhage since the 3 days of life; her electroencephalogram showed a suppression burst pattern and brain MRI was normal; the concentration of glycine in CSF and in serum, and the CSF/serum ratio were 270 umol/L, 880umol/L and 0.31, respectively. At 16 days of age, sodium benzoate and dextromethorphan were administered. Imipramine was initiated at 7 years of age. *In vitro* expression analyses showed that the mutant glycine decarboxylases with p.N150T had 1% normal enzyme activity. However, the patient had far better psychomotor development because the other mutation, p.R790W, retained 14% of the enzyme activity (16). Kure et al. reported another Asian patient with p.N150T and c.1926 + 1G>A mutations (14). Her glycine level in CSF and the CSF/serum ratio were 148 mM and 0.12, respectively. No other phenotype was described. For residue 421 of glycine decarboxylase, variations of p.G421V and p.A729Efs^*^3 have already been described as associated with NKH (3). No other clinical data are available.

Currently, there are no formal management guidelines for NKH. The current treatment strategy focuses on reducing the plasma glycine concentration, blocking N-methyl-D-aspartate receptors, and symptomatic care (17, 18). Treatment information is available for six patients with severe NKH previously described in the literature (Table 1). Among three cases treated with dextromethorphan and/or sodium benzoate, one died at 4 months (case 2 in Table 1) and two were alive with severe intellectual disability when followed up at 1 year 7 months (case 1 in Table 1) and 7 months (case 9 in Table 1), respectively. The remaining three cases treated only with symptomatic care died from 4 to 13 days of life. Although information about the treatment provided was not available for the two siblings with attenuated NKH (case 5 and case 6 in Table 1), valproate administration leading to aggravation of the condition in the early stage of the disease was reported for case 5. Based on this, valproate is contraindicated for controlling epileptic seizures in NKH patients (19). In addition, based on previous case reports, vigabatrin should be avoided to treat West syndrome in NKH (20).

Since the genetic basis of NKH is well-defined and increased residual GCS activity has been associated with an improved clinical outcome, therapeutic strategies that can enhance the residual activity associated with a mutation should be pursued. For example, a translational read-through inducer has been used to promote translation through a premature stop codon (21), chemical chaperones have been used for unstable missense mutations to restore protein folding (22), and antisense oligonucleotides have been used to correct a splicing variation (23). Considering that CSF samples of neonatal children are not easily obtained, genetic testing is a powerful tool for diagnosing NKH, especially for patients with a normal plasma glycine level (case 2 in Table 1). Early use of genetic testing to diagnose NKH can avoid unnecessary drug-induced damage, as well as provide guidance to the family for future pregnancies.

## Conclusion

We reported a patient with severe NKH who carried a novel compound heterozygous mutation and summarized the genetic and phenotypic characteristics, as well as the treatment strategy followed and prognosis of other NKH cases reported in China to date. This case report highlights the importance of genetic testing not only as a tool for NKH diagnosis but also for clinical outcome prediction, and the potential development of novel therapeutic strategies in the future.

## Data Availability Statement

The original contributions presented in the study are included in the article/supplementary materials, further inquiries can be directed to the corresponding author/s.

## Ethics Statement

The studies involving human participants were reviewed and Hebei Province. Written informed consent to participate in this study was provided by the participants' legal guardian/next of kin. Written informed consent was provided by the participants' legal guardian/next of kin for the publication of this case report.

## Author Contributions

YC wrote the manuscript. LM, YZ, JJ, and WP followed-up the patient and conducted data collection. LM conceived the study and supervised this research. All authors performed critical reading and approved the final version of manuscript.

## Conflict of Interest

The authors declare that the research was conducted in the absence of any commercial or financial relationships that could be construed as a potential conflict of interest.

## Publisher's Note

All claims expressed in this article are solely those of the authors and do not necessarily represent those of their affiliated organizations, or those of the publisher, the editors and the reviewers. Any product that may be evaluated in this article, or claim that may be made by its manufacturer, is not guaranteed or endorsed by the publisher.

## References

[1] Toone JR Applegarth DA Coulter-Mackie MB James ER. Recurrent mutations in P- and T-proteins of the glycine cleavage complex and a novel T-protein mutation (N145I): a strategy for the molecular investigation of patients with nonketotic hyperglycinemia (NKH). Mol Genet Metab. (2001) 72:322–5. 10.1006/mgme.2001.3158 11286506

[2] Van Hove JLK Coughlin CII Swanson M Hennermann JB. Nonketotic hyperglycinemia. 2002 Nov 14 [Updated 2019 May 23]. In: Adam MP Ardinger HH Pagon RA Wallace SE Bean LJH Mirzaa G editors. GeneReviews® [Internet]. Seattle (WA): University of Washington, Seattle. 1993–2021. 20301531

[3] Coughlin CR 2nd Swanson MA Kronquist K Acquaviva C Hutchin T Rodríguez-Pombo P. The genetic basis of classic nonketotic hyperglycinemia due to mutations in GLDC and AMT. Genet Med. (2017) 19:104–11. 10.1038/gim.2016.74 27362913

[4] Kure S Takayanagi M Narisawa K Tada K Leisti J. Identification of a common mutation in Finnish patients with nonketotic hyperglycinemia. J Clin Invest. (1992) 90:160–4. 10.1172/JCI115831 1634607 PMC443076

[5] Liu S Wang Z Liang J Chen N Ou Yang H Zeng W. Two novel mutations in the glycine decarboxylase gene in a boy with classic nonketotic hyperglycinemia: case report. Arch Argent Pediatr. (2017) 115:e225–e9. 10.5546/aap.2017.eng.e225 28737873

[6] Gao Z Jiang Q Chen Q Xu K. Clinical and molecular genetic study of nonketotic hyperglycinemia in a Chinese family. Zhongguo Dang Dai Er Ke Za Zhi (Chinese). (2017) 19:268–71. 10.7499/j.issn.1008-8830.2017.03.003 28302194 PMC7390153

[7] Dai H Cai L Wu Z. A neonate with nonketotic hyperglycinemia. Neural Injury And Functional Reconstruction (Chinese). (2017) 12:18. 10.16780/j.cnki.sjssgncj.2017.02.033

[8] Cheng L Song T Li Y Xu H Xu Y Wan S. Identification and analysis of a novel mutation of glycine decarboxylase gene in a neonata with nonketotic hyperglycinemia. Chinese Journal of Perinatal Medicine (Chinese). (2017) 20:527–9. 10.3760/cma.j.issn.1007-9408.2017.07.010

[9] Jiang T Jiang J Xu J Zhen J Jiang P Gao F. Clinical and genetic analyses of a family with atypical nonketotic hyperglycinemia caused by compound heterozygous mutations in the GLDC gene. Zhongguo Dang Dai Er Ke Za Zhi (Chinese). (2017) 19:1087–91. 10.7499/j.issn.1008-8830.2017.10.011 29046206 PMC7389275

[10] Lin Y Zheng Z Sun W Fu Q. A novel compound heterozygous variant identified in GLDC gene in a Chinese family with non-ketotic hyperglycinemia. BMC Med Genet. (2018) 19:5. 10.1186/s12881-017-0517-1 29304759 PMC5755286

[11] Kanno J Hutchin T Kamada F Narisawa A Aoki Y Matsubara Y. Genomic deletion within GLDC is a major cause of non-ketotic hyperglycinaemia. J Med Genet. (2007) 44:e69. 10.1136/jmg.2006.043448 17361008 PMC2598024

[12] Xiong HY Alipanahi B Lee LJ Bretschneider H Merico D Yuen RKC. RNA splicing. The human splicing code reveals new insights into the genetic determinants of disease. Science. (2015) 347:1254806. 10.1126/science.1254806 25525159 PMC4362528

[13] Swanson MA Coughlin CR Jr Scharer GH Szerlong HJ Bjoraker KJ Spector EB. Biochemical and molecular predictors for prognosis in nonketotic hyperglycinemia. Ann Neurol. (2015) 78:606–18. 10.1002/ana.24485 26179960 PMC4767401

[14] Kure S Kato K Dinopoulos A Gail C DeGrauw TJ Christodoulou J. Comprehensive mutation analysis of GLDC, AMT, and GCSH in nonketotic hyperglycinemia. Hum Mutat. (2006) 27:343–52. 10.1002/humu.20293 16450403

[15] Conter C Rolland MO Cheillan D Bonnet V Maire I Froissart R. Genetic heterogeneity of the GLDC gene in 28 unrelated patients with glycine encephalopathy. J Inherit Metab Dis. (2006) 29:135–42. 10.1007/s10545-006-0202-6 16601880

[16] Kure S Ichinohe A Kojima K Sato K Kizaki Z Inoue F. Mild variant of nonketotic hyperglycinemia with typical neonatal presentations: mutational and in vitro expression analyses in two patients. J Pediatr. (2004) 144:827–9. 10.1016/j.jpeds.2004.02.044 15192636

[17] Hamosh A McDonald JW Valle D Francomano CA Niedermeyer E Johnston MV. Dextromethorphan and high-dose benzoate therapy for nonketotic hyperglycinemia in an infant. J Pediatr. (1992) 121:131–5. 10.1016/S0022-3476(05)82559-4 1385627

[18] Bjoraker KJ Swanson MA Coughlin CR 2nd Christodoulou J Tan ES Fergeson M. Neurodevelopmental outcome and treatment efficacy of benzoate and dextromethorphan in siblings with attenuated nonketotic hyperglycinemia. J Pediatr. (2016) 170:234–9. 10.1016/j.jpeds.2015.12.027 26749113

[19] Hall DA Ringel SP. Adult nonketotic hyperglycinemia (NKH) crisis presenting as severe chorea and encephalopathy. Mov Disord. (2004) 19:485–6. 10.1002/mds.10681 15077252

[20] Tekgul H Serdarolu G Karapinar B Plat M Yurtsever S Tosun A. Vigabatrin caused rapidly progressive deterioration in two cases with early myoclonic encephalopathy associated with nonketotic hyperglycinemia. J Child Neurol. (2006) 21:82–4. 10.1177/08830738060210011801 16551461

[21] Sánchez-Alcudia R Pérez B Ugarte M Desviat LR. Feasibility of nonsense mutation readthrough as a novel therapeutical approach in propionic acidemia. Hum Mutat. (2012) 33:973–80. 10.1002/humu.22047 22334403

[22] Majtan T Liu L Carpenter JF Kraus JP. Rescue of cystathionine beta-synthase (CBS) mutants with chemical chaperones: purification and characterization of eight CBS mutant enzymes. J Biol Chem. (2010) 285:15866–73. 10.1074/jbc.M110.107722 20308073 PMC2871454

[23] Hua Y Sahashi K Hung G Rigo F Passini MA Bennett CF. Antisense correction of SMN2 splicing in the CNS rescues necrosis in a type III SMA mouse model. Genes Dev. (2010) 24:1634–44. 10.1101/gad.1941310 20624852 PMC2912561

